# Approximating the Hotelling observer with autoencoder-learned efficient channels for binary signal detection tasks

**DOI:** 10.1117/1.JMI.10.5.055501

**Published:** 2023-09-26

**Authors:** Jason L. Granstedt, Weimin Zhou, Mark A. Anastasio

**Affiliations:** a University of Illinois Urbana-Champaign, Department of Computer Science, Champaign, Illinois, United States; b Shanghai Jiao Tong University, Global Institute of Future Technology, Shanghai, China; c University of Illinois Urbana-Champaign, Department of Bioengineering, Champaign, Illinois, United States

**Keywords:** objective image quality assessment, numerical observers, channelized Hotelling observer, autoencoder, neural networks

## Abstract

**Purpose:**

The objective assessment of image quality (IQ) has been advocated for the analysis and optimization of medical imaging systems. One method of computing such IQ metrics is through a numerical observer. The Hotelling observer (HO) is the optimal linear observer, but conventional methods for obtaining the HO can become intractable due to large image sizes or insufficient data. Channelized methods are sometimes employed in such circumstances to approximate the HO. The performance of such channelized methods varies, with different methods obtaining superior performance to others depending on the imaging conditions and detection task. A channelized HO method using an AE is presented and implemented across several tasks to characterize its performance.

**Approach:**

The process for training an AE is demonstrated to be equivalent to developing a set of channels for approximating the HO. The efficiency of the learned AE-channels is increased by modifying the conventional AE loss function to incorporate task-relevant information. Multiple binary detection tasks involving lumpy and breast phantom backgrounds across varying dataset sizes are considered to evaluate the performance of the proposed method and compare to current state-of-the-art channelized methods. Additionally, the ability of the channelized methods to generalize to images outside of the training dataset is investigated.

**Results:**

AE-learned channels are demonstrated to have comparable performance with other state-of-the-art channel methods in the detection studies and superior performance in the generalization studies. Incorporating a cleaner estimate of the signal for the detection task is also demonstrated to significantly improve the performance of the proposed method, particularly in datasets with fewer images.

**Conclusions:**

AEs are demonstrated to be capable of learning efficient channels for the HO. The resulting significant increase in detection performance for small dataset sizes when incorporating a signal prior holds promising implications for future assessments of imaging technologies.

## 1 Introduction

Medical imaging systems are commonly optimized with consideration of a specific task.^1^ Assessing the performance of such systems requires an objective metric for image quality.^2^^–^^6^ For signal detection tasks, the Bayesian ideal observer (IO) has been advocated for producing a figure-of-merit for assessing imaging systems because it can maximize the amount of task-specific information in the measurement data.^2^^–^^7^ For a binary signal detection task, the IO test statistic takes the form of a likelihood ratio. Using this likelihood ratio as a test statistic in turn maximizes the area under the receiver operating characteristics (ROC) curve.^2^^–^^4^^,^^7^ However, analytically determining the IO is generally difficult because it typically is a nonlinear function and requires complete knowledge of the statistical properties of the image data.

There has been recent progress in developing approximations for computing the IO test statistic.^5^^,^^6^ One line of research involves sampling-based methods that utilize Markov-chain Monte Carlo methods to approximate the IO, but the work in this area has largely been limited to relatively simple stochastic object models.^3^^,^^5^^,^^8^^,^^9^ Another development is the approximation of the IO with convolutional neural networks (CNNs).^10^^–^^12^ Generative adversarial models have also been employed to approximate the IO with Markov-Chain Monte Carlo methods.^13^^,^^14^ An alternative method to approximating the IO’s performance employs variational Bayesian inference.^15^ This line of research has shown promise for implementing task-specific optimization of sparse reconstruction methods.

A common surrogate for the often-intractable IO is the Hotelling observer (HO).^16^^–^^19^ The HO is the optimal linear discriminator for maximizing the signal-to-noise ratio (SNR) of the test statistic.^20^^,^^21^ Directly implementing the HO requires the estimation and inversion of a covariance matrix, which quickly grows as image size increases and can become intractable to compute.^22^ There are a few different strategies for mitigating the computational cost of inverting a large matrix.^16^^,^^22^ One method is to avoid a direct inversion by implementing an iterative approach to estimate the test statistic.^2^ If the measurement noise covariance matrix is known and an estimate of the background covariance matrix is available, covariance matrix decomposition is another option,^2^ with the caveat that certain situations can lead to significant bias in the performance.^23^ Alternatively, the test statistic can be learned directly from the images provided that there is a sufficient amount of data.^11^^,^^24^ The most commonly employed method, however, is the implementation of channels that approximate the HO.^5^^,^^25^^,^^26^ Additionally, a recent comparison between nonlinear CNN-based methods and linear channelized methods for simple signals on simulated digital mammography (DM) images found that channelized methods can outperform CNN-based methods when training data are limited.^27^ Conceptually, channels function by projecting the high-dimensional image data to a low-dimensional image manifold.^28^^–^^30^ Ideally, the manifold embedding would preserve the important features of the data.^31^ This dimensionality reduction stabilizes the process of estimating the HO when considering noisy data and limited amounts of training images.

Channelized observers are considered efficient if they closely approximate the original observer’s performance.^32^ Prior work in computing efficient channels includes Laguerre–Gauss (LG),^26^ singular value decomposition (SVD),^33^ the filtered channel observer (FCO),^34^ and partial least squares (PLS).^35^ Although some methods generate generic channels that can be applied to classes of signals,^26^ the more recent work has focused on channels that are applicable to a specific detection task.^35^^,^^36^ These channels require a dataset of images for training but are applicable to detection tasks where the signal is not known exactly or rotationally symmetric.^35^^,^^36^ In addition to learning efficient channels, there are approaches that seek to mimic the human observer’s performance.^37^^–^^39^ An early approach learned the relationship between channel features and human observer performance with a support vector machine.^37^ Another approach investigated optimization with respect to the HO and human observers on accelerated MRI reconstruction.^38^ There has also been some recent work applying CNNs for directly learning a surrogate measure of human observer performance.^39^ In the remainder of this work, however, the focus is on efficient channels.

Autoencoders (AEs) are a type of artificial neural network (ANN) that are characterized by a mirror structure, with the target output of the network similar to the input.^40^^–^^44^ They are designed to learn a lower-dimensional representation of the data called an embedding. The portion of the network that transforms the input to the embedding is known as the encoder, and the portion that transforms the embedding back into the original data space is known as the decoder. A good embedding is capable of significant data compression while retaining most of the information from the original data. The data compression qualities of AEs make them desirable to use in many tasks, and they have been applied in state-of-the-art systems for classification,^45^ noise reduction,^46^ and regression.^47^ The widespread success of the AE is due to its ability to generate low-dimensional representations of images, which increases the efficiency of further processing by attenuating noise and embedding data to its most important components. Linear AEs share some similarities with principle component analysis. In general, a linear AE with optimal weights projects the data onto a subspace spanned by the top principle directions of the data.^48^

In this work, the problem of learning task-informed embeddings with an AE is explored to develop efficient channelized observer methods. AEs have previously been demonstrated to develop efficient channels for simple signal-known-exactly/background-known-statistically (SKE/BKS) detection tasks.^36^^,^^49^ This paper expands on those works by demonstrating that the learning task for an AE is equivalent to learning channels for the HO. The AE is modified to learn the optimum transformation matrix that maximizes the amount of task-specific information encoded in its latent states. Experiments are performed to validate AE-learned channels on location-known binary detection tasks, including SKS tasks with an elliptical signal on a lumpy background and detection tasks with two separate signals on a breast phantom background. The performance of the AE-learned channels in these studies is compared to state-of-the-art channelized methods, including PLS and FCO, as well as conventional methods for estimating the HO. A study is also performed to assess the ability of these channelized methods to generalize to related imaging systems. Finally, the impact of the quality of the signal estimate on channelized methods is explored.

The remainder of this work is organized as follows. In Sec. 2, the salient aspects of binary signal detection theory are presented. The HO, channelized HO (CHO), and AE are also reviewed in that section. A methodology for learning channels for a location-known binary signal detection task using an AE is developed in Sec. 3. The numerical studies and results of the proposed method for approximating the HO are included in Secs. 4 and 5, along with a comparison to other state-of-the-art methods. Finally, this paper concludes with a discussion of the work in Sec. 6.

## 2 Background

We consider a linear digital imaging system described as

g=Hf(r)+n, (1)

where f(r) represents the object function with the spatial coordinate $r\in R^{K}$, H represents a continuous-to-discrete (C-D) imaging operator that maps $L_{2}(R^{K})\to R^{N}$, $n\in R^{N}$ is the random measurement noise, and $g\in R^{N}$ denotes the measured image data vector. Hereafter, the object function f(r) will be abbreviated as f.

### 2.1 Overview of Binary Signal Detection Tasks

The binary signal detection task considered involves the classification of an image by an observer into one of two hypotheses: signal-present ($H_{1}$) or signal-absent ($H_{0}$). Under these hypotheses, the imaging process can be represented as

$$H_{0}:\;g=Hf_{b}+n,$$ (2a)

$$H_{1}:\;g=H(f_{b}+f_{s})+n,$$ (2b)

where $f_{b}$ and $f_{s}$ denote a background and a signal object, respectively. Depending on the imaging task, these can be either random or fixed.

In the binary signal detection task, a real-valued test statistic t(g) is obtained from applying an observer to a measured image. This test statistic is compared against a threshold τ to classify g as satisfying either $H_{0}$ or $H_{1}$. To determine the desired performance for the signal detection task, an ROC curve can be plotted to depict the trade-off between the false-positive fraction and the true-positive fraction by varying the threshold τ. The overall signal detection performance of the observer can be summarized by computing the area under the ROC curve (AUC).^50^

### 2.2 Bayesian Ideal Observer and Hotelling Observer

The IO is optimal and sets the upper limit for observer performance on signal detection tasks. The IO test statistic for binary tasks is defined as a monotonic transformation of the likelihood ratio $\Lambda_{\mathrm{LR}}(g)$ that takes the form^2^^,^^3^^,^^12^

$$\Lambda_{\mathrm{LR}}(g)=\frac{p(g|H_{1})}{p(g|H_{0})},$$ (3)

where $p(g|H_{0})$ and $p(g|H_{1})$ are conditional probability density functions that describe g under the hypotheses of $H_{0}$ and $H_{1}$, respectively.

An alternative to the IO for assessing signal detection performance is the HO. The HO test statistic is defined as

$$t(g)=w_{\mathrm{HO}}^{T}g,$$ (4)

where $w_{\mathrm{HO}}\in R^{M}$ denotes the observer template. Let ${\overset{¯}{g}}_{j}\equiv {\left\langle {\left\langle g\right\rangle }_{g|f}\right\rangle }_{f|H_{j}}$ denote the conditional mean averaged with respect to object randomness and noise associated with $H_{j}$. The Hotelling template $w_{\mathrm{HO}}$ is defined as^2^

$$w_{\mathrm{HO}}={\left[\frac{1}{2}(K_{0}+K_{1})\right]}^{-1}\Delta \overset{¯}{g},$$ (5)

where

$$K_{j}={\left\langle {\left\langle [g-{\overset{¯}{g}}_{j}]{\left[g-{\overset{¯}{g}}_{j}\right]}^{T}\right\rangle }_{g|f}\right\rangle }_{f|H_{j}},$$ (6)

$$\Delta \overset{¯}{g}={\overset{¯}{g}}_{1}-{\overset{¯}{g}}_{0},$$ (7)

where $K_{j}$ and ${\overset{¯}{g}}_{j}$ represent the covariance matrix and mean of the measured data g under hypothesis $H_{j}$, respectively. The mean signal $\Delta \overset{¯}{g}$ is the difference of the means under the two hypotheses. A regularized Moore–Penrose (MP) pseudoinverse can be applied instead of the inverse in Eq. (5) in cases where the covariance matrix is ill-conditioned.^2^

The SNR associated with the test statistic t is another commonly employed FOM for assessing signal detection performance and is given by^21^

$${\mathrm{SNR}}_{t}=\frac{{\left\langle t\right\rangle }_{1}-{\left\langle t\right\rangle }_{0}}{\sqrt{\frac{1}{2}\sigma_{0}^{2}+\frac{1}{2}\sigma_{1}^{2}}},$$ (8)

where ${\left\langle t\right\rangle }_{j}$ and $\sigma_{j}^{2}=\langle {\left(t-{\left\langle t\right\rangle }_{j}\right)}^{2}\rangle_{j}$ are the mean and variance of t(g) under hypothesis $H_{j}$. The HO maximizes the SNR of its test statistic t(g), but unlike the IO it does not necessarily maximize the AUC.^21^

### 2.3 Channels

In some cases, the direct estimation of the HO can become intractable. The covariance matrix in Eq. (5) may not be invertible due to the size of the images or insufficient quantity of data. As images become larger, the amount of data required to obtain a robust estimate noticeably increases. Medical image datasets frequently do not contain sufficient images to robustly estimate $K_{j}$ due to large image sizes or the limited access to large scale annotated datasets.

To mitigate this problem, a channelized version of the image g can be introduced as^21^

v=Tg=T(Hf+n), (9)

where v is a M×1 channel-reduced image and T is a M×N matrix. The dimensionality reduction is determined by the number of channels M<N. Applying the HO to the channel-reduced data yields the CHO,^21^ with the test statistic taking the form

$$T(v)=w_{v}^{T}v={\left(K_{v}^{-1}\Delta \overset{¯}{v}\right)}^{T}v,$$ (10)

where $K_{v}=\frac{1}{2}[K_{v,1}+K_{v,0}]$ and $\Delta \overset{¯}{v}={\overset{¯}{v}}_{1}-{\overset{¯}{v}}_{0}$, where $K_{v,j}={\left\langle {\left\langle [v-{\overset{¯}{v}}_{j}]{\left[v-{\overset{¯}{v}}_{j}\right]}^{T}\right\rangle }_{v|f}\right\rangle }_{f|H_{j}}$ and ${\overset{¯}{v}}_{j}={\left\langle \langle v\rangle_{v|f}\right\rangle }_{f|H_{j}}$ for j=(0,1).

Efficient channels should maximize the retained task-relevant information to provide an efficient approximation of the HO. There are several methods that exist for selecting efficient channels. One of the first was LG channels.^32^ These channels are a combination of a Gaussian function with a Laguerre polynomial and were proposed due to their structural similarity with the Hotelling template for certain detection tasks. These channels are suitable for a smooth rotationally symmetric signal on a lumpy background but may have suboptimal performance for arbitrary signals and more complex backgrounds.^35^

An alternative to LG channels are SVD channels.^33^ These channels are singular vectors that form a basis for image vectors in the range of the imaging operator. The most efficient set of channels constructed from this method involved decomposing the noiseless signal image by use of the singular vectors and choosing the top m of them to form the channel set. However, this method is computationally expensive and system-specific.

Two current state-of-the-art methods for generating efficient channels capable of considering arbitrary signals and backgrounds without any specific knowledge of the imaging system are PLS^35^ and the FCO.^34^ The PLS method applies a data reduction technique that iteratively constructs a number of latent vectors that maximize the covariance between the data and a binary label representing the presence of a signal. The PLS method represents an attractive method to use in limited-data cases and/or large image sizes and works well with noisy and heavily correlated data. However, the technique can suffer from a degradation of performance when the amount of available image data is small.^35^

FCO channels were initially developed as anthropomorphic channels to approximate human signal detection performance for irregularly shaped signals.^34^ However, FCO channels have also been explored as efficient channels for the HO.^34^^,^^51^^,^^52^ The FCO convolves a selected set of baseline channels with the signal before computing the observer template. For the experiments in this paper, LG channels were selected as the baseline set of channels due to both LG’s past success^32^ and similar decisions with the FCO method in more recent work.^51^^,^^52^ This realization of the FCO method will be referred to below as a convolutional LG observer.

### 2.4 Neural Networks for Approximating the IO

A feed-forward ANN is a system of computational units associated with tunable parameters called weights.^53^^,^^54^ A feed-forward ANN is capable of approximating any continuous function if it has a sufficiently complex architecture.^55^^,^^56^ ANNs have been employed to form numerical observers, with the focus on directly estimating the test statistic.^10^^–^^12^ Kupinski et al.^12^ utilized conventional fully connected neural networks to approximate the IO on low-dimensional extracted image features. Zhou and Anastasio extended this work to higher-dimensional data and allowed for native processing of image data by replacing the FCNN with a CNN.^10^^,^^11^ However, these approaches focus on learning the test statistic directly and may require a complex network and a large amount of training data to accurately approximate the IO.

### 2.5 Autoencoders

A specialized type of ANN is the AE.^40^^–^^44^ The AE is characterized by a mirror structure, with the input of the network similar to the target output. An AE has three distinct components: an encoder, an embedding, and a decoder. The encoder transforms the input to the embedding, which generally has a significantly reduced dimensionality compared to the input. The decoder transforms the embedding into the target output. In a canonical AE, the decoder is specified to reconstruct an approximation of the input to the encoder. AEs are frequently employed for their data compression properties in state-of-the-art systems for classification,^45^ regression,^47^ noise reduction,^46^ anomaly detection,^57^ and image recovery^58^ tasks. Additional performance improvements can be made by injecting additional information into the AE training process. Studies have shown that exploiting *a priori* information via modifications to the loss function can introduce task-specific information in the training of AEs.^59^^,^^60^ In contrast to previous work with ANNs, an AE is usually trained in an unsupervised way.^45^

In general, the layers in an AE specify many sets of matrix multiplications with added bias terms and nonlinear transformations. By restricting the operations to only matrix multiplications, linear AEs can be obtained. In these cases, the encoder and decoder can each be described by a transformation matrix that transforms to or from the data embedding. Such a simplified network is considered in this work since this configuration’s encoder has a natural parallel with the channel matrix in the CHO. The input to the network is an image, and the target output is either the input image or a related version of the input image, depending on the task.

Another aspect of AEs that has recently been considered is the concept of tied weights.^61^ Tied weights further enforce the mirror-like structure of the AE by forcing the encoder and decoder matrices to be symmetric. Tied-weight AEs have been shown to perform similar to untied-weight AEs but require less data to train because of the reduction in parameters.

An optimization problem is solved to determine the weights of the AE by minimizing a reconstruction loss. Let $W_{1}$ and $W_{2}$ be N×M weight matrices that parameterize the encoder and decoder of the linear AE, respectively. The optimization problem to obtain the optimal weights of a linear AE is

W^1,W^2=arg minW1,W2‖W2W1Tg−g*‖2, (11)

where the target reconstruction is represented by $g^{*}$. This target reconstruction can be the same as or different from the input data but is usually closely related. For example, in denoising problems, the target output is a clean version of the input image.

The solution of the optimization problem is computed by minimizing the loss function using a variation of the backpropagation algorithm.^62^ The conventional loss function for an AE is the mean squared error between the input and the output of the network. Given P vectorized background images $g_{i}$, the conventional loss function corresponding to a zero-bias linear AE is^40^

$$L_{\mathrm{con}}(W_{1},W_{2})=\frac{1}{P}\overset{P}{\underset{i=1}{\sum }}{\left\|W_{2}W_{1}^{T}g_{i}-g_{i}^{*}\right\|}_{2}^{2}.$$ (12)

The data embedding of the trained AE is represented as

y^=W^1Tg, (13)

and the corresponding reconstruction is g^=W^2y^.

## 3 Method: Autoencoder-Learned Channels

A method for learning efficient channels for the CHO with an AE is described below. A connection between AE weights and the CHO framework is established to illustrate the connection between the learned data embeddings and more traditional channels.

### 3.1 Autoencoder Channels and Linear Autoencoders

The learned weights of an AE have an additional interpretation when considered in the framework of a signal detection task. The weights define a mapping from the high-dimensional image space to a low-dimensional embedding space. This is conceptually equivalent to the CHO channel matrix T. Thus the learned AE weights can be employed as channels for the CHO by setting $T=W_{1}^{T}$ in Eq. (9). Intuitively, these AE-learned channels capture the data most important for reconstructing the image. Combining a conventional AE with directly learning an observer on the data embedding has also been proposed.^49^ However, here AE channels with a conventional method for obtaining the CHO will be considered.

The embedding in Eq. (13) causes the AE to encode the entirety of the input image. This makes the conventional AE suboptimal for learning channels because a significant portion of the data embedding is dedicated to reconstructing certain components of the image that may not be highly relevant to the detection task. To circumvent this, as described below, information about the signal can be incorporated into the AE training process to preserve task-specific information.

### 3.2 Task-Specific Autoencoders

**Fig. 1:**
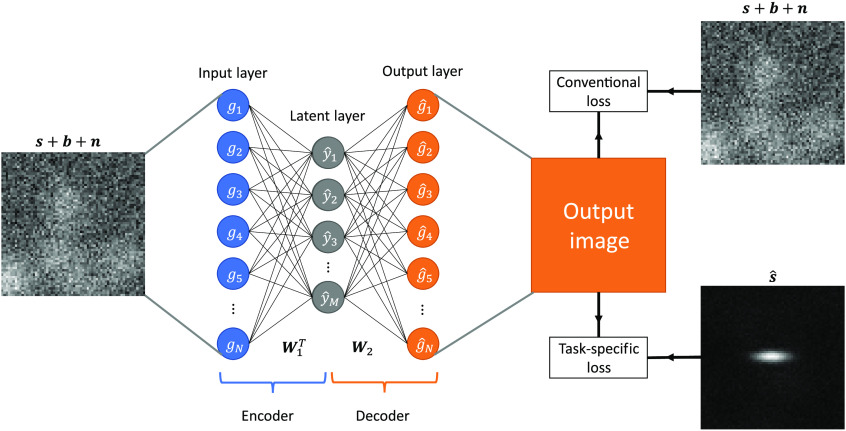
Diagram of the proposed method. A noisy image is the input to an encoder, which is mapped to an M-dimensional latent space to encode the information. The encoder transformation matrix is given by $W_{1}^{T}$. The embedded representation is then multiplied by the decoder transformation matrix $W_{2}$ to return to image space and generate the output image. Two different loss functions are considered for training this model. The conventional loss function computes the MSE between the output image and the input image. This approach attempts to reconstruct the entire input image. The second considered loss function is the task-specific loss, which calculates the difference between the output image and estimated signal image s^. This loss maximizes the signal-specific information of the input image.

**Fig. 2:**
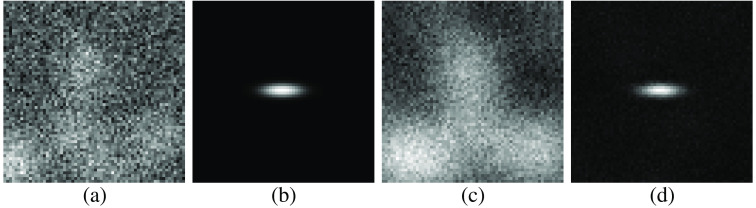
Reconstructed images corresponding to each of the loss functions. The grayscale in each case is adjusted to maximize visibility. Panel (a) is the input image, which contains the faint Gaussian elliptical signal in (b). The conventional AE with 20 channels reconstructs the image in (c) while the task-specific tied-weight AE with 10 channels reconstructs the image in (d). Note that the reconstructed image in (c) is noticeably less noisy than the input image (a), which it is attempting to reconstruct. This is due to the limited number of latent states in the model embedding the largest structures in the input images. Noise cannot be effectively encoded for an image, so it is attenuated.

A modification to the loss function to improve the learned data embedding and resulting signal detection performance for AE-channels is presented here. Ideally, the entirety of the AE embedding would be dedicated to the task-specific information. This would minimize the proportion of the embedding that is dedicated to extraneous information and lead to a more efficient set of channels. By changing the AE’s target reconstruction to the mean signal image for a binary signal detection task, the background and noise are suppressed during the reconstruction process. This results in an embedding in which the signal can be accurately represented. This substitution of the target reconstruction minimizes the MSE between the reconstructed image and the estimated signal image and takes the form of

$$L_{\text{task}}(W_{1},W_{2})=\frac{1}{P}\overset{P}{\underset{i=1}{\sum }}{\left\|W_{2}W_{1}^{T}g_{i}-I(g_{i})\Delta \overset{¯}{g}\right\|}_{2}^{2},$$ (14)

where $\Delta \overset{¯}{g}$ is defined in Eq. (7) and I(·) is the indicator function that returns 1 if the signal is present and 0 otherwise. Since the loss function employs label information, this approach is a supervised learning method. Considering the background as noise to be suppressed permits the entire capacity of the embedding to focus on the task-specific information. Although a linear AE projects data into a subspace spanned by the data’s top principle directions,^48^ the proposed modification removes this association. Using the signal template as the target image assists the training process in identifying an embedding that preserves task-specific information. As shown below, this modification to the loss function is capable of generating efficient channels for the CHO. A diagram of the AE with both the conventional and task-based approach for the signal detection task is provided in Fig. 1, with a sample reconstruction from AEs trained using both loss functions shown in Fig. 2. Both the task-specific and conventional loss functions can be minimized by use of a gradient-descent method, with specific implementation details provided in Sec. 4.6.

## 4 Numerical Studies

Numerical simulation studies were conducted to evaluate the performance of the proposed method for learning efficient channels for the CHO. All simulations addressed BKS signal detection tasks. Four distinct binary signal detection tasks were considered. Using a lumpy stochastic object model, a location-known task and a fixed-centroid signal-known-statistically task were considered. These tasks enabled the HO to be estimated both using covariance matrix decomposition^2^ and direct computation according to Eq. (5). These observers will be referred to as HO-CMD and HO-Direct, respectively. On a breast phantom background, two location-known signal detection tasks using signals of different shapes and sizes were considered. These tasks allowed for the evaluation of channelized methods on a more realistic medical imaging task. ROC curves were fit by use of a binormal model^50^^,^^63^^,^^64^ with the fitted AUC values reported. The experimental results are reported in distinct sections based on the image background model, with the details for each signal detection task and the training of neural networks are given in the appropriate sections.

### 4.1 Signal Detection Tasks That Utilize a Lumpy Background Model

Two different signal detection tasks were performed with consideration of a lumpy stochastic object model^65^ with an idealized parallel-hole collimator system.^3^ Further details about each of the components are provided below.

#### 4.1.1 Lumpy background

**Fig. 3:**
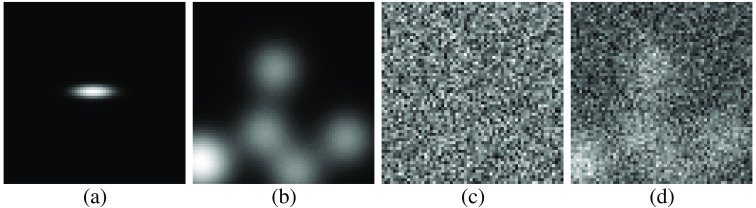
Sample generation for signal-present images used in the lumpy background experiments. The grayscale in each case was adjusted to maximize visibility. The signal image (a) was added to the lumpy background (b) and the Gaussian noise (c) to produce the composite dataset image (d). The signal image is an elliptical Gaussian signal with width $\sigma_{x}=5$, $\sigma_{y}=1.5$.

A stochastic lumpy object model was used as the background^65^

$$f_{b}(r)=\overset{L}{\underset{n=1}{\sum }}l(r-r_{n}|a,s),$$ (15)

where $L∼\text{Poiss}(\overset{¯}{L}=5)$ is the number of lumps that is sampled from Poisson distribution with the mean set to 5 and $l(r-r_{n}|a,s)$ is the lump function modeled by a symmetric 2D Gaussian function with amplitude a and width s given by

$$l(r-r_{n}|a,s)=a\;\exp(-\frac{\left(r-r_{n}{)}^{T}(r-r_{n}\right)}{2s^{2}}),$$ (16)

where $r_{n}$ is the uniformly sampled position of the n’th lump. The magnitude and width of the lumps were set to the frequently employed values of a=1 and s=7.^10^^,^^35^^,^^49^^,^^65^ An example of a signal-present image in the dataset with a circular signal is located in Fig. 3.

#### 4.1.2 Imaging system

The stylized imaging system in these studies was a linear C-D mapping describing an idealized parallel-hole collimator system with a point response function given by^3^^,^^66^

$$h_{m}(r)=\frac{h}{2\pi w^{2}}\;\exp(-\frac{{\left(r-r_{m}\right)}^{T}(r-r_{m})}{2w^{2}}),$$ (17)

with the height h=40, the width w=0.5, and the location of the m’th pixel in r denoted by m.

#### 4.1.3 Signals

The signal function $f_{s}$ was a 2D Gaussian function:

$$f_{s}(r)=A\;\exp(-(R_{\theta }(r-r_{c}){)}^{T}D^{-1}(R_{\theta }(r-r_{c}))),$$ (18)

where A=0.2 is the amplitude and $r_{c}$ is the coordinate of the signal location. Here $R_{\theta }$ is the Euclidean rotation matrix that rotates the Gaussian by an angle of θ and is given by

$$R_{\theta }=[\begin{array}{cc} \cos\;\theta & -\sin\;\theta \\ \sin\;\theta & \cos\;\theta \end{array}],$$ (19)

and D is a scaling matrix that controls the width of the Gaussian along each axis and is given by

$$D=[\begin{array}{cc} 2\sigma_{x}^{2} & 0\\ 0 & 2\sigma_{y}^{2} \end{array}].$$ (20)

For both experiments involving the lumpy background, the elliptical Gaussian signal was set to have the parameters $\sigma_{x}=5$ and $\sigma_{y}=1.5$. The image size was selected to be 64×64 with the signal centered at $r_{c}={\left[32,32\right]}^{T}$. The value of θ varied depending on the type of task.

#### 4.1.4 Detection tasks

The first signal detection task considered a single orientation of the signal by setting θ=0. The signal template was computed according to Eq. (7), which resulted in a noisy estimate of the signal. The second signal detection task sampled θ uniformly from the set [0 deg,45 deg,90 deg,135 deg]. This allowed for four distinct orientations of the elliptical Gaussian. The mean signal was also computed with Eq. (7), which resulted in a noisy estimate of the signal averaged across the four possible realizations.

#### 4.1.5 Dataset generation

A training set of 60,000 unique background images with noise were generated for the lumpy object model. The background images were generated separately from the signal image in Eq. (18) using the appropriate background model. Each background image was summed with a unique noise vector drawn from an independent and identically distributed Gaussian distribution $n_{m}∼N(0,\delta^{2})$ with a mean of 0 and standard deviation δ=20. These images were then paired, with half designated for signal present and half for signal absent. Each signal present image was summed with the signal image to generate the final training dataset of 30,000 paired images. Another set of 5000 paired images was generated for determining the channel covariance matrix after the channels had been learned and a further set of 5000 paired images were held out as a testing dataset.

### 4.2 Location-Known Tasks That Utilize a Breast Phantom Dataset

Two additional signal detection tasks were performed with consideration of a breast phantom stochastic object model employing the VICTRE dataset.^51^^,^^52^ This dataset contains simulated DM images and was employed previously in a location-known mathematical observer study to evaluate imaging systems.^51^^,^^52^ The images were divided into four categories of breast types: extremely dense, heterogeneously dense, scattered fibroglandular, and fatty. The signals in the dataset were microcalcification clusters and spiculated masses. Regions of interest (ROIs) from the simulated DM image were extracted to generate the images used in the signal detection task. For each signal, there were associated signal-absent and signal-present images. The spiculated mass ROIs were 109×109  pixels and the microcalcification ROIs were 65×65  pixels. The considered microcalcification signal was a consistent arrangement of five microcalcifications, which were considered jointly as one signal for the detection task. The signal remained constant throughout all the signal-present images, but a clean signal image was assumed to be unavailable. An estimation of the signal was obtained from the difference of the mean signal-present and signal-absent images according to Eq. (7), making this a location-known task.^51^^,^^52^

**Fig. 4:**
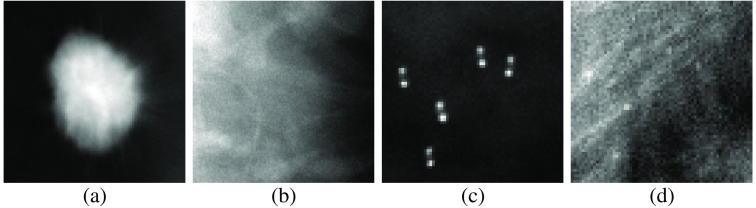
Sample estimated signals and images from the VICTRE breast phantom dataset. The grayscale was adjusted in each case to maximize visibility. Panels (a) and (c) contain the mean signal image of the spiculated mass and microcalcification cluster, respectively. Panels (b) and (d) are sample images of those corresponding signals embedded into a fatty breast phantom. Note that the signal in (c) appears to be doubled. This artifact is a result of the extracted scattered breast ROIs not aligning with the other breast types in the provided public dataset.

For each type of signal, 12,500 total images were selected from the dataset to form training, validation, and testing sets of 5000, 625, and 625 paired images, respectively. The breast types selected maintained the proportions employed in the VICTRE study.^51^^,^^52^ The signals were estimated by taking the mean of the signal-present images and subtracting the mean of the signal-absent images for the combined training and validation dataset. Sample images and estimated signals are included in Fig. 4.

### 4.3 Location Known Tasks Considering a Provided Signal

The tasks in Secs. 4.1 and 4.2 consider a mean signal that is computed separately for each dataset size. This represents the case where the training data are used to estimate the mean signal. However, one can also consider the performance of methods when the mean signal is provided. This represents cases where a prior about the signal is known and can be leveraged to improve detection performance. Such experiments isolate the contribution of the mean signal estimate from additional images provided by larger datasets. This experiment formulation has also been employed previously to evaluate the dependence of channelized methods on the quantity of background images.^35^ The experiments in Secs. 4.1 and 4.2 were repeated, setting the mean signal to be the estimate from the training and validation data at the maximum dataset size for cases.

### 4.4 Tasks Evaluating the Generalizability of Channelized Methods

Since the proposed method of determining channels is dependent on the mean signal image, there is the reasonable concern that AE-learned channels may fail to generalize outside of the learned dataset. Thus two studies were considered to evaluate the ability of the learned channelized methods to generalize.

#### 4.4.1 Domain shift study

The first case considers the effects of domain shift on the channels. Domain shift is the difference in distributions between the datasets an algorithm is trained and evaluated upon.^67^ A method’s robustness to domain shift corresponds to generalization performance when a set of channels developed for one imaging system is employed in another related system with different properties. Such a circumstance may occur when using channels to tune an imaging system’s parameters, as it is undesirable to recompute channels whenever part of the system is changed. Characterization of such performance is important when considering the application of channels developed with consideration of one imaging system to data produced by another imaging system. Medical imaging datasets are frequently difficult to obtain, and it may be beneficial to leverage previously collected datasets to develop channels for use with new imaging systems.

An experiment was developed to investigate the impact of domain-shift on the learned channels. To evaluate observer performance in the presence of domain shift, a source dataset and a target dataset are necessary. The source dataset is defined as the training dataset for a channelized method. Likewise, the target dataset is the dataset upon which the channelized method is evaluated. The difficulty of the problem depends on the difference in the distributions between the source and target dataset.

To evaluate generalization performance across multiple instances of domain shift, three datasets were constructed and all possible combinations of source and target datasets were considered. The imaging system, location-known elliptical signal, and stochastic object model for the background were reused from Sec. 4.1.1. These parameters correspond with a location-known detection task for a stylized PET imaging system. The three datasets were constructed by setting the width of the imaging system in Eq. (17) to 1.0, 2.0, and 4.0. Thus the domain shift is the difference in image distributions from stylized PET imaging systems with different widths.

The noise model was also altered from the previous studies. A mixed Poisson/Gaussian noise model was considered, as it is known to be a reasonable approximation of PET noise.^68^ Each pixel of the image was sampled as a random Poisson variable with the mean set to the intensity of the voxel and combined with additive i.i.d. Gaussian noise. This noise model takes the form of

$$n_{m}=p_{m}+\sigma_{m},$$ (21)

where $p_{m}∼\text{Poiss}(g_{m})$ and $\sigma_{m}∼N(0,1)$. After adding the noise, the images were scaled to [0,1].

The generated datasets had training, validation, and testing sizes of 30,000, 5000, and 5000 paired signal present/absent images, respectively. The same set of backgrounds were considered for each dataset.

#### 4.4.2 Amalgamated dataset study

Due to the difficulty of obtaining medical imaging data and the benefits of additional data for improving performance, it is sometimes beneficial to consider related images acquired from multiple imaging conditions. For this combined dataset to be beneficial for application across multiple imaging conditions, the imaging conditions must be similar enough such that the benefits of the additional data outweigh the loss of imaging-condition specific channels. The performance of the obtained channels upon the individual imaging conditions that contributed to the combined dataset is relevant for evaluating the benefits of such a technique.

To evaluate such a case, pairs of images were evenly sampled from the three constructed datasets in Sec. 4.4.1 to generate a fourth dataset. Backgrounds were sampled without replacement and selected from train/validation/testing sets independently to avoid contamination. This dataset served as an additional source and was denoted as the amalgamated dataset. The performance of channels trained on the amalgamated dataset was evaluated on each of the three component datasets to simulate deploying the learned channels to each of the contributing imaging conditions.

### 4.5 AE Topology

The considered network topology was a tied-weight AE with no nonlinear or bias terms. This structure parallels the CHO formulation in Eq. (9), as the AE is learning the transformation matrix T. Tied weights were chosen because they couple the encoder and the decoder by enforcing $W_{1}^{T}=W_{2}$, making the encoder a transpose of the decoder. This formulation prevents loss of information that may solely exist in the decoder since only the encoder is employed as the transformation matrix. Additionally, tied weight AEs have fewer parameters to train and thus are expected to perform better in limited-data experiments.^36^

### 4.6 Experimental Parameters

#### 4.6.1 Training details

AE-channels were determined by minimizing the modified AE loss function in Eq. (14). The models were trained in Tensorflow^69^ using the Adam algorithm.^62^ The AE weights were initialized using a truncated normal initializer with a standard deviation of $5\times 10^{-6}$. The models were trained for 500 epochs. In cases where the considered dataset contained more than 500 images, pre-training the models on a subset of 500 images for 500 epochs to burn in the network sometimes improved performance. A mini-batch size of 250 was employed, with an equal number of signal-present and signal-absent images in each mini-batch. The learning rate was determined empirically and set to $5\times 10^{-3}$ for the VICTRE phantom background study and $1\times 10^{-5}$ for the lumpy background study. All networks were trained on a single NVIDIA TITAN X GPU.

Several channelized reference methods were implemented to compared against the AE-learned channels, including convolutional LG,^34^ PLS,^35^ and the nonprewhitening matched filter (NPWMF).^21^ The HO-Direct^21^ was also computed on each subset using Eq. (5). The MP pseudoinverse was employed instead of the traditional inverse in these cases. Regularization was applied to the MP pseudoinverse in the form of truncating scaled singular values below $1\times 10^{-6}$, with the cutoff point determined empirically. A grid search on the entire training dataset for each background was performed for each dataset size to select the parameters for all methods, with the maximum number of channels capped at 20. This grid search also implicitly provided multiple random initializations for the AE.

#### 4.6.2 Evaluation

Subsets of the training data were considered to evaluate the performance of models as the amount of available data varied. The VICTRE experiments detailed in Sec. 4.2 contained subsets of size K=250, 500, 1000, 2000, and 5000 image pairs. The larger lumpy background experiments detailed in Sec. 4.1 also considered sets of 10,000, 15,000, 20,000, 25,000, and 30,000 image pairs. The generalization and amalgamation studies considered a single case of 30,000 images.

The standard train-validate-test scheme^70^ was employed to evaluate performance. The AE and competing methods were given the training data and signal estimate to operate on, with the performance evaluated on the validation data to select the best set of parameters. For all methods, the mean signal image used during training was computed from the difference of the signal present and signal absent training images. Once the parameters were determined for each method, the CHO was numerically determined according to Eq. (10). The final models were then evaluated on the testing set to obtain the AUC values.

The HO-CMD was also computed for the experiments on lumpy backgrounds to analyze the efficiency of the channels for each method. This method has been demonstrated to approximate the HO when noiseless images are available and the noise model is known,^2^ forming an upper bound for the experiments and permitting the evaluation of the efficiency of the channelized methods. The empirical background covariance matrix was calculated using the combined training and validation datasets for a total of 70,000 noiseless background images. This method was unavailable for estimating the HO of the VICTRE experiments as noiseless images were not available.

## 5 Results

**Fig. 5:**
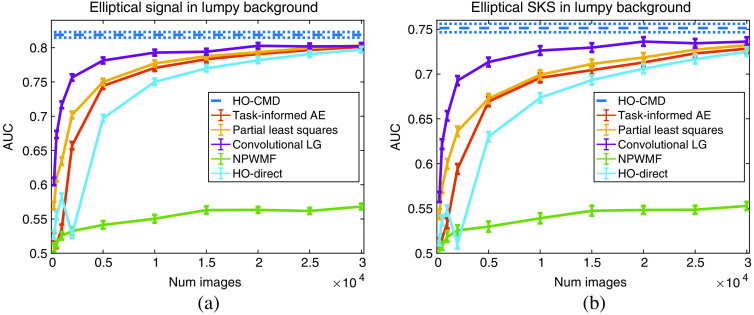
Performance of the CHO on varying training dataset sizes for the lumpy background model. Panel (a) contains the results for the location-known elliptical signal while (b) contains the SKS elliptical signal results. The error bars correspond to the standard deviation of the fit AUC values. The HO-CMD is provided as an estimate of the upper bound of the HO, given an infinite amount of images, and is included to benchmark the efficiency of the channels for all methods.

**Fig. 6:**
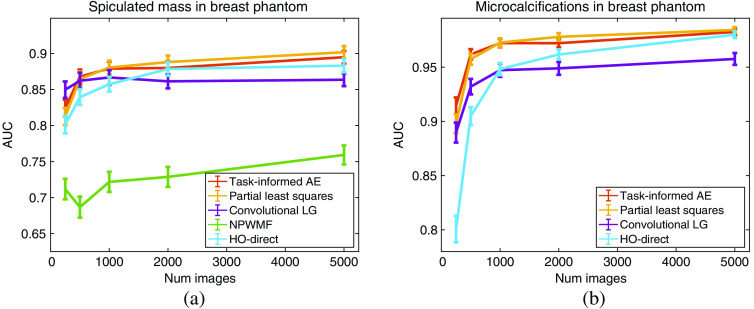
Performance of the CHO on varying training dataset sizes for the VICTRE breast phantom model. Panel (a) contains the results for the spiculated mass signal while (b) contains the microcalcification cluster results. The error bars correspond to the standard deviation of the fit AUC values. The analytic HO estimate is not included as the clean images are not available to generate an estimate. The NPWMF is also omitted in (b) since it has an AUC below 0.53 for all considered dataset sizes, significantly less than the other methods.

**Fig. 7:**
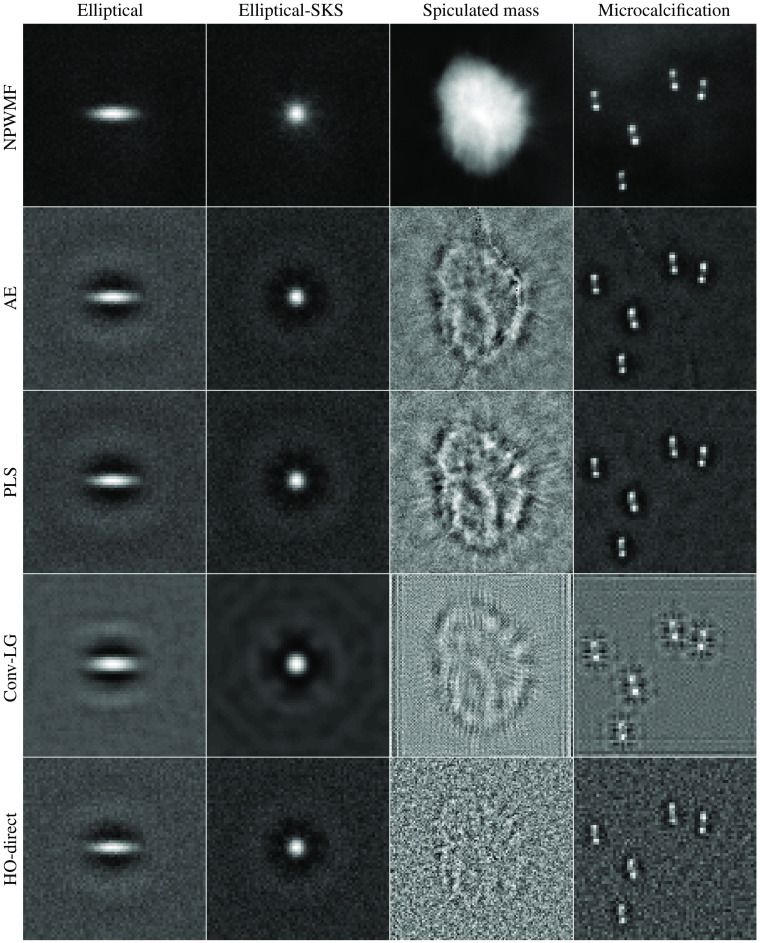
Visualization of the observer templates obtained from each of the channelized methods for the considered backgrounds and signals. Each column contains results for a given signal/background combination and each row contains the observer templates for a specific channelized method.

**Fig. 8:**
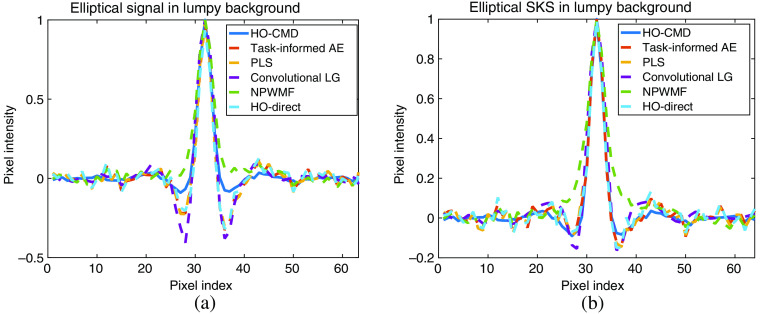
Lineplots of the considered observers for the lumpy background model on the 30,000 image training dataset. The lineplot is a diagonal cross section of the observer template from the upper left to the lower right. Panel (a) contains the results for the location-known signal while (b) contains the SKS elliptical results.

The results for the limited-image tests for the lumpy model and VICTRE breast phantom model are provided in Figs. 5 and 6. A visualization of the generated observer templates for each of the considered methods at the maximum number of considered images is included in Fig. 7, with a diagonal cross-section of the some of the observers contained in Fig. 8. Overall, the proposed task-informed AE method was competitive with the state-of-the-art channelized methods for both the lumpy background and VICTRE phantom background cases. For the lumpy background cases, the best performing model was convolutional LG. This is likely due to the smooth, symmetric signal supporting LG channels. The task-informed AE and PLS methods produced AUC values that were within statistical error of each other at most of the considered dataset sizes. The HO-Direct had inferior performance to all channelized methods while also requiring significantly more computation to evaluate. Thus some channelized methods outperformed the standard method of computing the HO. The HO-CMD serves as an upper bound. As the amount of training data increases, it is expected that the channelized methods will approach the HO-CMD provided that sufficient channels are available since the CHO is a special case of the HO. However, this was not investigated as it requires significant amounts of data and is not a practical use case.

A few additional architectures were considered. The first modification to the architecture was replacing the decoder of the AE with a fully connected neural network and minimizing a sigmoid cross-entropy loss employing the image label rather than a mean image. This modification had inferior performance when compared to the proposed method. One potential reason for this is less information available for training the method—one label as opposed to a full signal image to compute the MSE. Previous works have demonstrated that it is possible to closely approximate the HO when employing a label instead of an image, but a more sophisticated loss function must be considered.^11^^,^^49^^,^^71^ This architecture is also incompatible with the experiment proposed in Sec. 4.3. A second modification was adding a ReLU function after the encoder to introduce a nonlinear function into the training process. This modification required the model weights to be untied for the training process to converge. This architecture also had inferior performance to the proposed method. A potential reason for this is the untied weights. The AE learns to leverage the nonlinear function to improve the signal image reconstruction and lower the loss function, but when the weights of the AE are used as channels in the CHO they no longer benefit from the nonlinear function. The symmetry between the AE training method and the CHO discussed in Sec. 3 is broken. Finally, the conventional AE was also tested but failed to exceed 0.55 AUC in all four experiments.

**Fig. 9:**
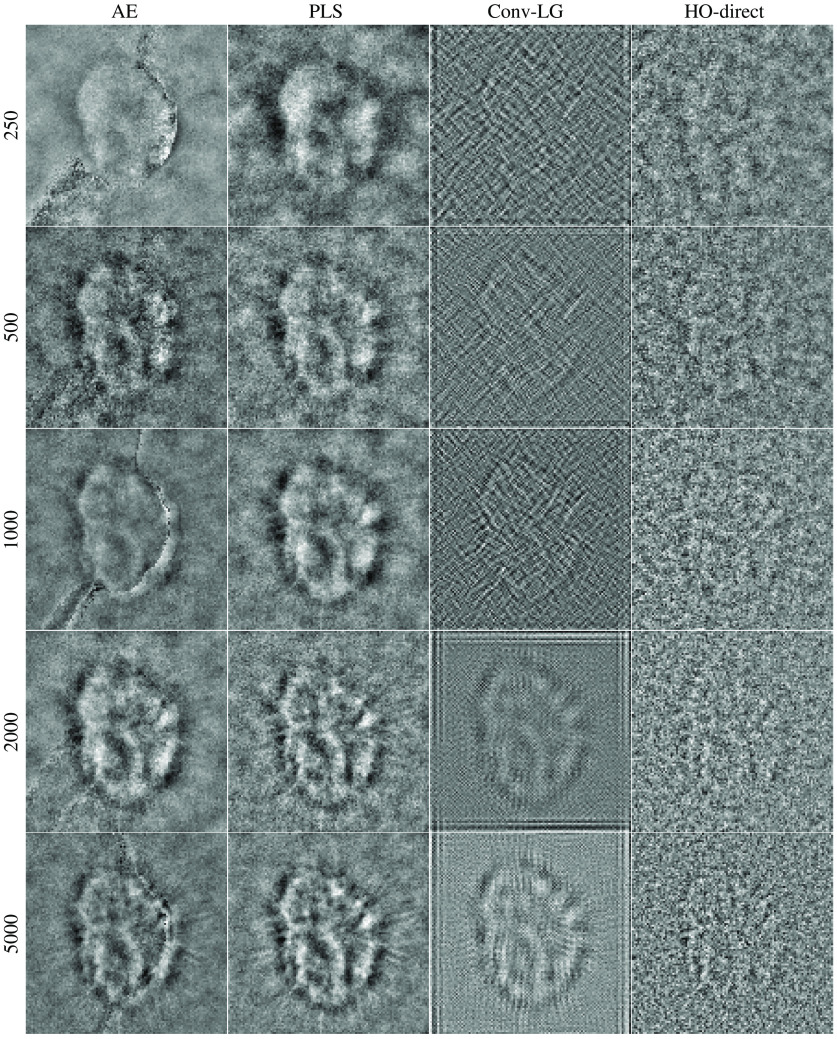
Observer templates associated with the VICTRE spiculated mass experiment. Each column contains one of the considered channelized methods and the rows containing the size of the associated training dataset.

In the VICTRE background case, the PLS and task-informed AE methods maintained their trend of the produced channels resulting in AUCs within statistical error of each other. However, in this case, both methods performed noticeably better than convoluational LG. The evolution of the channels for the spiculated mass as the number of images are increased is in Fig. 9. There was a noticeable seam in the observer templates for the task-informed AE channels on the phantom data, which is a remnant of the random initialization of the training method. This seam does not significantly affect observer performance, which may be why it was not removed in the training process. An additional contributing factor to the emergence of this seam may be the more complicated, nonsymmetric signal. Compared to the lumpy background experiments, the more complex VICTRE signal likely contributes to the substandard performance of the convolutional LG method. In addition, the increased structure complexity of the signal and the relatively higher noise are likely contributing factors in the earlier plateau of observer performance in the VICTRE case compared to the lumpy background experiments.

**Fig. 10:**
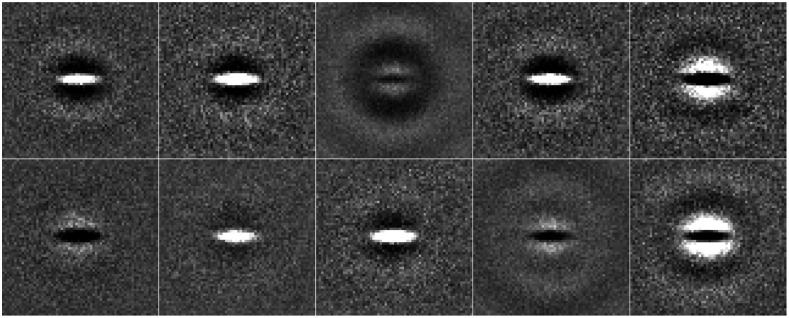
Location-known elliptical channels associated with the AE-learned HO template for the 30,000 paired image dataset on the lumpy background. The grayscale is constant and fixed. The channels are ordered left to right, top to bottom. The ordering of the channels was determined through an ablation study.

**Fig. 11:**
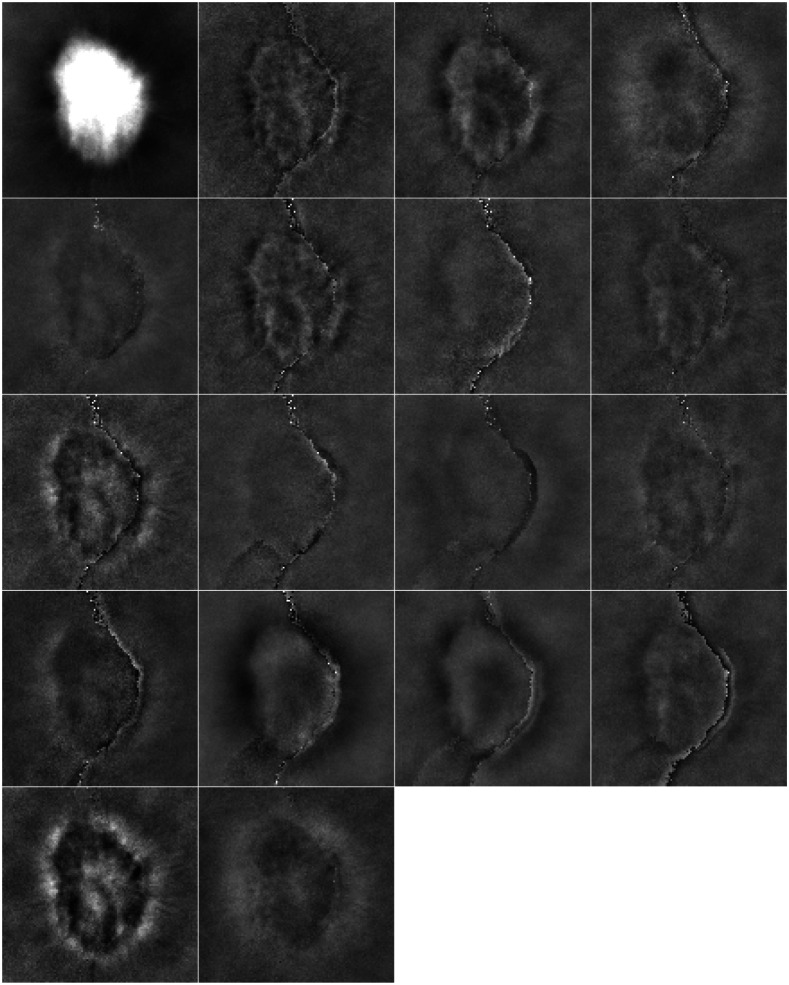
Eighteen channels associated with the AE-learned HO template for the VICTRE spiculated mass detection task, displayed on the range [−0.01,0.01]. The channels are ordered left to right, top to bottom. The ordering of the channels was determined through an ablation study. Note the presence of a seam running through several of the channels. This seam does not significantly impact observer performance and is likely a result of the stochastic learning process and more complicated structure of the signal.

The learned channels for the 30,000 location-known lumpy image case and the 5000 VICTRE spiculated mass case are included in Figs. 10 and 11, respectively. Many of the channels are similar to one another in the features they extract and can be removed without significant loss of performance. These extraneous channels likely exist due to the AE training process. Random initializations generate different starting locations for each channel, which is iteratively optimized by the AE training process. During this process, the channels are updated to better jointly reconstruct the signal image. Thus even if the final model makes inefficient use of its full channel budget, the channels are influenced by their interactions during the training process. One of the limitations of this approach is its sensitivity to the random initialization, which can result in models of dramatically varying quality even with the same structure.

**Fig. 12:**
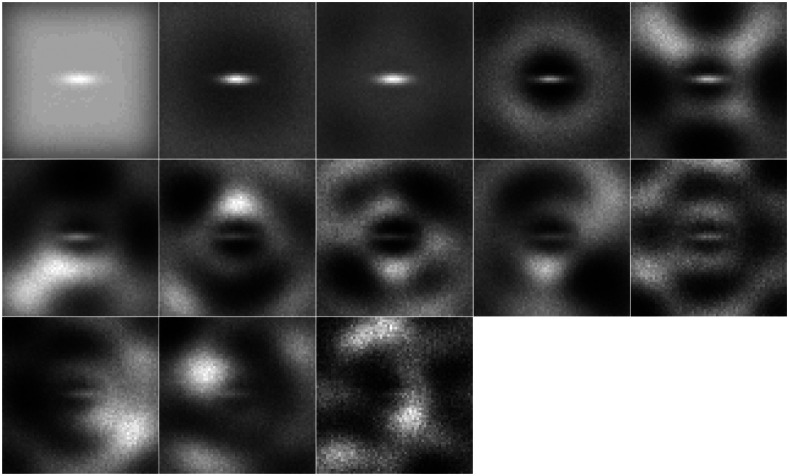
Location-known elliptical channels associated with PLS for the 30,000 paired image dataset on the lumpy background. The grayscale is constant and fixed. The channels are ordered left to right, top to bottom. The channels are presented in the order produced by the iterative PLS method.

**Fig. 13:**
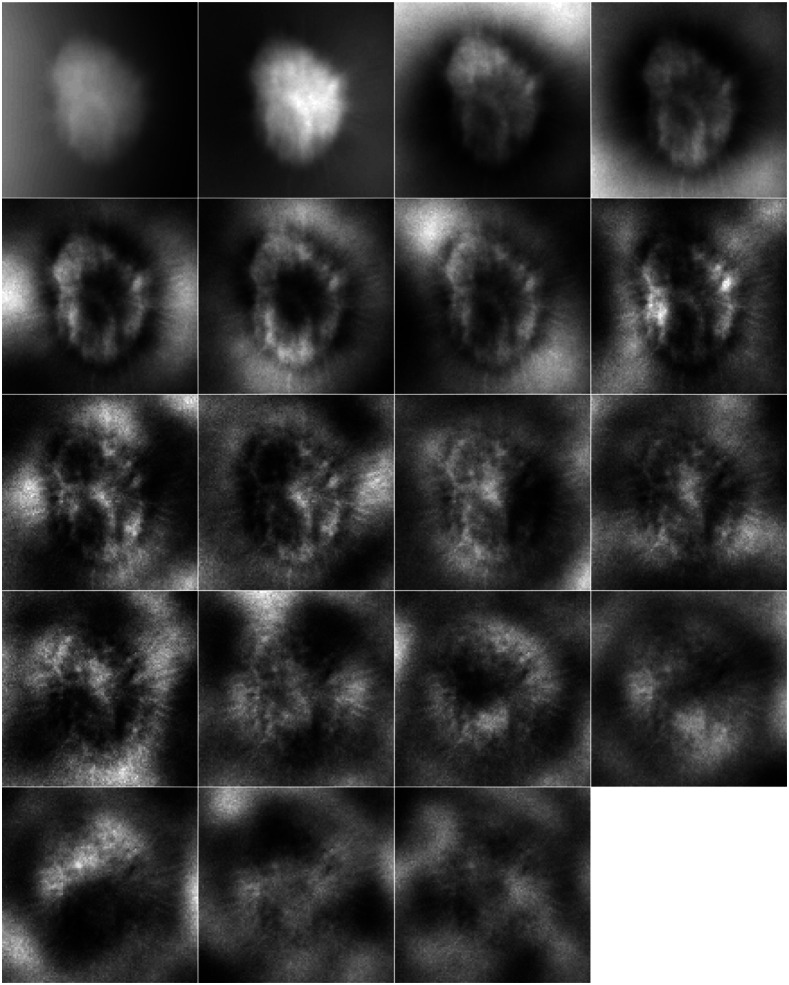
Ninteen channels associated with the PLS HO template for the VICTRE spiculated mass detection task, displayed on the range [−0.01, 0.01]. The channels are ordered left to right, top to bottom. The channels are presented in the order produced by the iterative PLS method.

In all four of the considered detection tasks, the performance of the AE-learned channels most closely followed the PLS channels. The PLS filters for the elliptical and spiculated mass signals are included in Figs. 12 and 13. In contrast to the AE-learned channels, there is a distinct pattern for each of the considered filters. Thus while the performance of the AE-learned channels scales approximately the same as PLS with additional images, the channels the two methods generate are quite distinct.

**Fig. 14:**
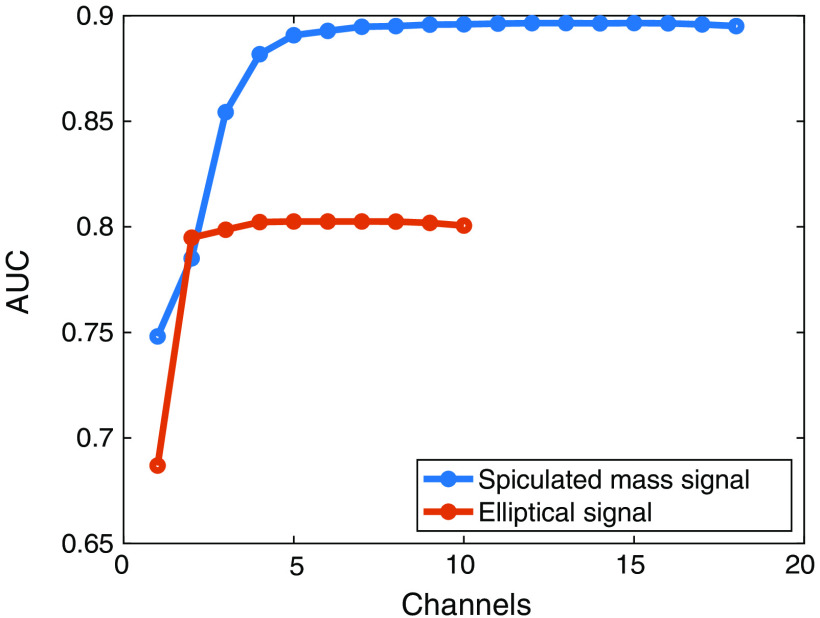
Results of the ablation study performed for the elliptical and spiculated mass experiments. The channel contributing the least was iteratively pruned to produce a rank ordering of the channels. A plateau occurs after a certain number of channels, depending on the signal complexity.

Since the AE training process simultaneously updates all channels, there is no natural ordering for the importance of the channels. To evaluate the contributions of each channel provided to the overall AUC, an ablation study was performed for the AE-learned channels for the elliptical signal with 30,000 training images and the spiculated mass signal with 5000 training images. The channel that contributes the least was iteratively pruned to form a rank ordering of the importance of the channels. The results of the ablation study are in Fig. 14. Although the first few channels contribute significantly to the performance of the observer, the results quickly plateau. The plateau occurs more slowly with the more complex spiculated mass signal, indicating that signal complexity impacts the minimum number of beneficial channels. Beyond this point, the random initializations provided by sweeping over a range of the channels likely matter more than the additional channel budget.

**Fig. 15:**
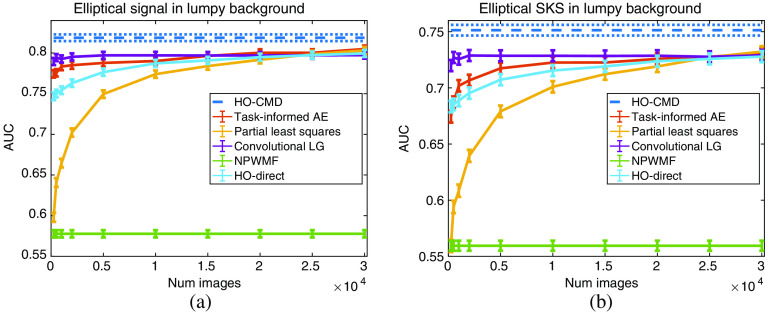
Performance of the CHO on varying training dataset sizes for the lumpy background model with a fixed mean signal estimated from the training and validation datasets. Panel (a) contains the results for the location-known elliptical signal while (b) contains the SKS elliptical signal results. The error bars correspond to the standard deviation of the fit AUC values. The HO-CMD is provided as an estimate of the upper bound of the HO, is given an infinite amount of images, and is included to benchmark the efficiency of the channels for all methods.

The results for the fixed-signal lumpy background experiments are in Fig. 15. For the lumpy background cases, the task-informed AE channels performed significantly better than the PLS channels for all but the largest dataset sizes. In those cases, performance was comparable. Convolutional LG channel performance was relatively static since the models were tuned at the maximum dataset size, and it is not a learning method but were the best performing channels for the majority of the lumpy dataset sizes considered. However, both the PLS and task-informed AE channels outperformed convolutional LG when sufficient images were available. Compared to the standard experiments in Fig. 5, the performance of the task-informed AE and convolutional LG methods significantly improve and the PLS method retains approximately the same curve. This performance increase is especially notable for the smaller dataset sizes.

**Fig. 16:**
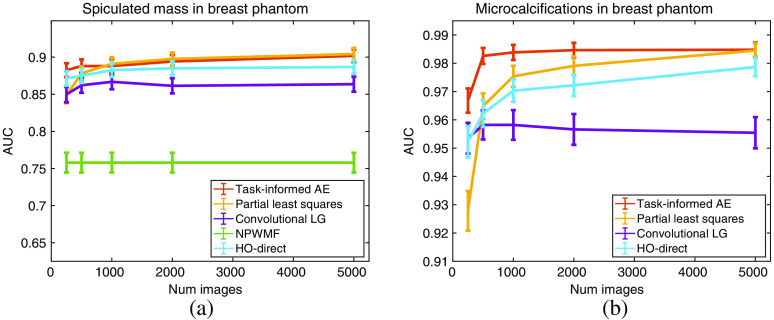
Performance of the CHO on varying training dataset sizes for the VICTRE breast phantom model with a fixed mean signal estimated from the training and validation datasets. Panel (a) contains the results for the spiculated mass signal while (b) contains the microcalcification cluster results. The error bars correspond to the standard deviation of the fit AUC values. The analytic HO estimate is not included as the clean images are not available to generate an estimate. The NPWMF is also omitted in (b) since it has an AUC below 0.53 for all considered dataset sizes, significantly less than the other methods.

In the fixed-signal VICTRE background case, contained in Fig. 16, the task-informed AE-learned channels outperform every other tested method for the smaller training subsets. Given a sufficient amount of data, the AE and PLS channels approach the same AUC and are approximately equivalent. This occurred more quickly for the larger spiculated mass signal than the smaller microcalcification clusters. Compared to the fixed-signal lumpy background experiments, the performance of the observers on the VICTRE data plateaued more quickly. This is likely due to the relatively higher noise in the lumpy background experiments and the more complicated signal in the VICTRE case. The significant boost in the performance of the AE method when a cleaner signal image is available demonstrates the potential of the method to leverage prior information when it is available. Compared to the standard case in Fig. 6, the AE and convolutional LG methods again demonstrate improvement with the better signal estimate, whereas the PLS method’s performance remains consistent. Overall, the ablation study results indicate that the proposed method is more sensitive to the quality of the mean signal than the amount of training images. This characteristic is shared with the FCO method but not PLS.

Although the ROI varied across the considered tasks, it did not appear to be a dominant factor in determining when the plateau occurred in observer performance. In particular, the spiculated mass detection task with the larger ROI had its AUC plateau more quickly than the simple elliptical signal with a smaller ROI. Despite the number of weights in the AE scaling with the size of the ROI, the statistical properties of the detection task are likely the determining factor for the amount of training data required. Thus the number of images required to reach the plateau is likely connected to the difficulty of the signal detection task. For the comparison of the spiculated mass and elliptical signal tasks, the difference in difficulty is most likely due to the magnitude of noise.

**Fig. 17:**
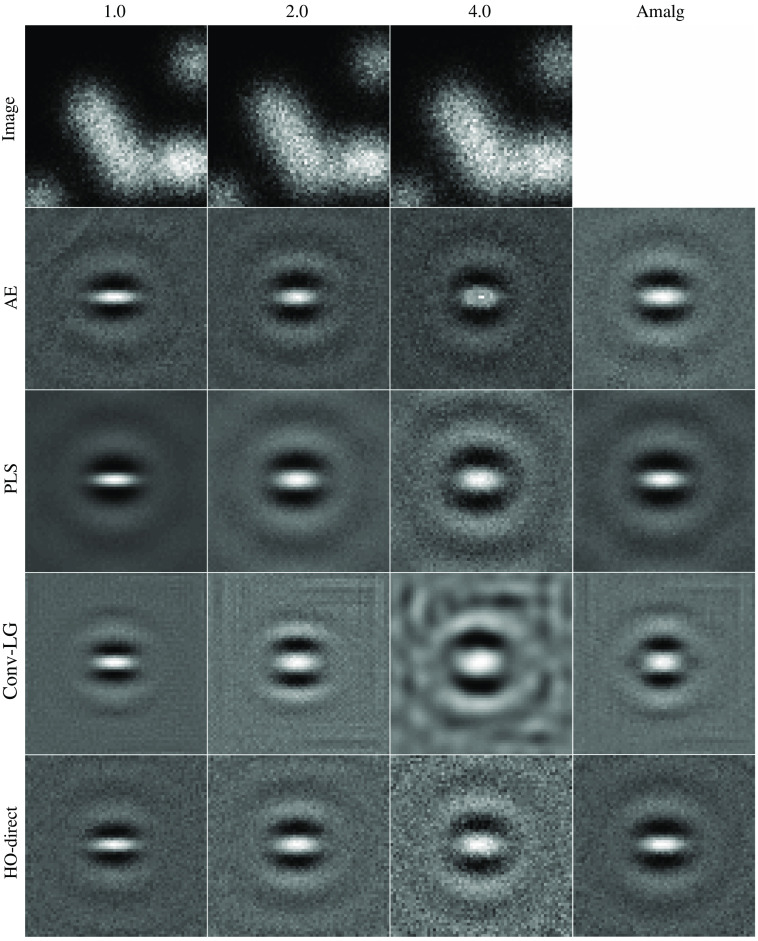
Sample images and templates from the generalization and amalgamation studies. The columns contain the results from varying the width of the imaging system, increasing from left to right. The first row contains a sample signal absent image from the dataset. The remaining rows contain the obtained observer templates for the considered methods.

The results for the domain shift and amalgamation studies are included in Table 1. Visualizations of the observer templates obtained for each of the considered width values are included in Fig. 17. For the 2.0 and 4.0 source system widths, the channels obtained from the task-informed AE generalized well to target imaging systems with widths both above and below the source system. The improvement was especially noticeable when applying the channels obtained from the 4.0 imaging system width data to the 1.0 imaging system width target images. In the 1.0 source imaging system width case, the channels from the task-informed AE were competitive when applied to data from the target imaging systems of 2.0 and 4.0 widths and outperformed both Conv-LG and HO-Direct channels, but PLS channels produced superior results. This indicates that the learned channels possess some measure of generalizability and resilience to errors in the employed signal image, despite the dependence of the proposed method on the estimate of the signal. PLS-generated channels demonstrated unusual behavior in that they generalized well to imaging systems with more blur but had noticeably inferior performance to the other considered methods when evaluated on imaging systems with less blurring. This is noticeable in the case with the channels obtained from the 1.0 source imaging system width generalizing the best of the considered methods to the 4.0 target system. However, PLS also performed the worst out of the observers when considering the channels obtained from the 4.0 source imaging system data to the 1.0 target imaging system, with the performance of PLS lagging the performance of the task-informed AE channels by 0.08 AUC.

**Table 1:** Results from the domain shift and amalgamation studies. Observers for each method were first obtained from each source system. Each of these observers were then applied to each target imaging system to evaluate their performance in the presence of domain shift. The source amalg case refers to the even sampling of the source system datasets to generate an amalgamated dataset, which was evaluated in the same way for each target imaging system. The standard error for all of the values is ±0.005. The highest values that are statistically significant for each of the target imaging systems within a source image system width are in bold.

Source system width	Observer method	Target system width		
		1.0	2.0	4.0
1.0	Task-informed AE	0.99	0.91	0.68
	PLS	0.99	**0.92**	**0.71**
	Conv LG	0.99	0.90	0.64
	HO-Direct	0.99	0.90	0.64
2.0	Task-informed AE	**0.99**	**0.92**	**0.71**
	PLS	**0.99**	**0.92**	**0.71**
	Conv LG	0.98	0.91	0.69
	HO-direct	**0.99**	**0.92**	0.70
4.0	Task-informed AE	**0.93**	**0.88**	**0.73**
	PLS	0.85	0.80	0.72
	Conv LG	0.89	0.85	**0.73**
	HO-direct	0.92	0.87	**0.73**
Amalg	Task-informed AE	**0.98**	**0.92**	0.73
	PLS	0.96	0.90	**0.74**
	Conv LG	**0.98**	0.91	0.71
	HO-direct	0.97	**0.92**	**0.74**

During the course of the experiments, it was observed that the convolutional LG channels were especially sensitive to the quality of the estimated signal. When provided with the signal used to generate the data in both the location-known and SKS lumpy experiments, the method outperformed all other competitors. When fewer images were used to generate the mean signal image and thus there is more noise in the signal image, such as in the VICTRE phantom dataset, the performance degraded significantly. This is illustrated in the dichotomy between standard and fixed signal results in Figs. 5 and 15. In the fixed signal case, the performance of the method barely changes as the number of paired images sweep from 500 to 30,000. Thus the performance improvement in the standard case is almost entirely due to the increase in the quality of the mean signal image. This is also visible in the observer templates constructed using convolutional LG channels, as they differ significantly from the templates constructed via other channelized methods. Although the task-informed AE-learned channels attempt to reconstruct the given signal image directly, and thus would seem to be impacted more by noise, the method was more robust to error in the estimated signal than the convolutional LG approach. Denoising AEs is known to reduce noise in an image since the AE embedding’s limited dimensionality lacks the capacity to represent the high-frequency components of the noise. The reduced sensitivity of the proposed method to minor variations in the data may be a result of this property.

## 6 Discussion and Conclusion

This study demonstrated that AEs are capable of learning efficient CHO channels for both location known and fixed-centroid SKS signal detection tasks. Data embeddings and observer channels were demonstrated to be fundamentally related, with the task of optimizing a data embedding to preserve signal-specific information equivalent to determining an efficient channel selection for the CHO. Furthermore, the presented method of computing channels is capable of meeting or exceeding the performance of state-of-the-art methods on the investigated tasks.

Channels were learned for the CHO by minimizing the reconstruction loss of an AE. Modification of the AE loss function to focus only on task-specific information involving the signal was found to have a significant benefit over using the conventional AE loss. Empirical sweeps over the network topology revealed that the AE could efficiently approximate the HO for a wide range of cases utilizing comparable numbers of channels to other approaches. The proposed method was equivalent to state-of-the-art approaches for the lumpy and VICTRE backgrounds. When a higher-quality signal estimate was provided, the AE-channels demonstrated significantly higher performance and were superior to the compared channelized methods on the VICTRE breast phantom dataset containing complicated signals. This increase was especially notable on smaller dataset sizes. Additionally, ability of the channels to generalize to imaging systems with varying parameters was evaluated. On average, the proposed method outperformed the other considered channelized approaches in cross-imaging system performance despite the injection of the signal image during the training process. In terms of appearance and performance, the AE channels were closest to PLS channels and the performance of the two methods was typically within error. However, AE-learned channels were noticeably better at generalizing. The AE-learned channels were sensitive to the random initialization of the weights and frequently learned redundant channels. The training scheme can likely be further improved with a more robust approach to weight initialization.

Opportunities for future work include evaluating the AE-learned channels with a channelized IO and extending the formulation to both more sophisticated SKS cases and 3D input images. The channels should work directly for any standard Markov chain Monte Carlo method for estimating the IO. Although the current form of the loss function for learning AE-channels requires knowing the signal centroid, it could be generalized by considering convolutional AEs.^72^

Another aspect that can be further developed is the resilience of the method to deterministic errors in the employed signal image. For instance, the supplied mean signal might be offset or larger/smaller than the original image. Although the generalizability study explored this in a limited way, a more structured study would be beneficial for developing more resilient methods. A deterministic shift of the mean image may benefit from untying the encoder and decoder, for instance.

The superior performance of AE-learned channels on smaller datasets and medically realistic phantoms also expands the applicability of the method to real-world cases, and the method should be tested on experimental data to identify remaining challenges in tuning the AE. The proposed method may also be applied toward learning anthropomorphic channels by replacing the ground-truth labels with human-generated labels during the training process. Acquiring human labels is a complex task that has several different elements in the study design, depending on the specific detection task and desired use case.^73^^–^^76^

## Data Availability

Code and data for this paper are publicly available at https://github.com/jasonlg/AETSI.
